# Potassium Titanate Assembled Titanium Dioxide Nanotube Arrays Endow Titanium Implants Excellent Osseointegration Performance and Nerve Formation Potential

**DOI:** 10.3389/fchem.2022.839093

**Published:** 2022-01-25

**Authors:** Hang Zhao, Feng Liu, Yixin Yin, Shuhua Wang

**Affiliations:** ^1^ State Key Laboratory of Crystal Materials, Shandong University, Jinan, China; ^2^ Oral Implantology Center, Jinan Stomatological Hospital, Jinan, China

**Keywords:** potassium, TiO_2_ surfaces, osteogenesis, neurogenesis, fully functional interface

## Abstract

Titanium based materials have been widely applied in bone-tissue engineering. However, inefficient bone repair remains to be solved due to the lack of neural network reconstruction at the bone-implant interface. Herein, we propose a functional surface modification approach to promote neurogenesis. Using an electrochemical technique and a hydrothermal approach, a potassium titanate nanorod-decorated titanium oxide (K_2_Ti_6_O_13_-TiO_2_) nanotube array is constructed on the surface of titanium implants. The K_2_Ti_6_O_13_-TiO_2_ hybrid nanotube array on titanium implants can enhance the osteogenic differentiation of mesenchymal stem cells due to the special nanostructures of titanium oxide nanorods. Meanwhile, the release of potassium ions is able to accelerate the neural differentiation of neural stem cells. This study provides a new approach to promote neuralization on the surface of implants, which is promising for future applications in constructing a fully functional interface in bone repair.

## Introduction

As the most common material for bone implants, titanium and its alloys have been widely used as screws to fix bone fractures (Koons et al., 2020). Although titanium implants are safe and possess high strength, toughness, and biocompatibility, their biological inertness often causes grafts to loosen and even escapes from the surrounding original bone tissue (Williams, 2021). Therefore, titanium implants should be endowed with an osseointegration ability to form a strong interface between the titanium implant and surrounding bone (Branemark, 1983). To date, many approaches to modifying the surface of titanium have been suggested, some of which have demonstrated ideal osteogenesis properties and achieved a strong connection between the implant and surrounding bone tissue (Visentin et al., 2019; Yu et al., 2020). It is well known that nerves are an important part of the surrounding bone tissue. Skeletal neurobiology has been the subject of intense research over the past few years, and increasing evidence has revealed the crucial interaction between bone and nerves (Brazill et al., 2019). Peripheral nerves participate in bone development and repair by secreting transmitters and other factors. Meanwhile, bone supplies mechanical support to hold the nerves within its internal milieu (Wan et al., 2021). The bones and nerves coordinate closely to ensure normal physiological functioning. However, this harmony is disrupted in areas of bone fracture. For example, in musculoskeletal injuries caused by trauma or sports, bone fractures are accompanied by nerve damage. Neural damage in bone defect regions may affect the repair procedure and hinder the full functional restoration of bone tissue (Barbe et al., 2013). Therefore, constructing a multi-tissue and multicellular structure is vital for tissue repair.

Various strategies to improve the bioactivity of titanium by modifying the surface of titanium implants have been explored (Guo et al., 2021; Li et al., 2021). TiO_2_ has been widely used in the construction of bioactive titanium implant surfaces. Many studies have verified that TiO_2_ can accelerate the osteogenic differentiation of mesenchymal stem cells (MSCs), and the nanostructure of TiO_2_ nanoarrays can also induce osteogenesis without the aid of any bio or chemical factor (Oh et al., 2006). The investigation by our group verified that the TiO_2_ nanorod array promoted the osteogenic differentiation of MSCs better than polished TiO_2_ ceramic (Qiu et al., 2016). Furthermore, successful osteointegration has been confirmed at the animal level. Wang et al. used minipigs to evaluate bone remodeling as their physiological and anatomical characteristics are similar to those of humans (Wang et al., 2011). The results demonstrated that TiO_2_ implants significantly promoted osteoconductivity and osteointegration.

Although much progress has been made in bone fracture healing, most studies ignored nerve reconstruction at the interface, which would result in the inefficient bone repair and loss of bone function. To achieve the formation of nerves in the newly formed bone area, the functional surface of titanium should be able to enhance both osteogenic and neural differentiation. As mentioned above, TiO_2_ and nanostructures on the surface of titanium implants can regulate the osteogenic differentiation of MSCs. Notably, the incorporation of essential biological elements has been proven to be beneficial for activating the peripheral nervous system (Liu et al., 2021) and regulating bone regeneration (Li et al., 2020). Potassium is one of the most abundant cations in intracellular fluid and plays an essential role in cell function, particularly in excitable cells, such as muscles and nerves (Fortin et al., 2008; Wang et al., 2020). The cellular membrane potential can be determined using the potassium gradient across the cell membrane. Potassium ions play a significant role in regulating the migration, elongation, proliferation, and differentiation of stem cells, especially in neural stem cells (NSCs) (Bai et al., 2013). The number of neurons and axonal extension can be increased by increasing the extracellular concentration of potassium (McFarlane and Pollock, 2000; Bosch et al., 2004). Some reports have demonstrated that cortical neurons exhibit long-term potentiation (LTP), which is a phenomenon related to synaptic plasticity after potassium ion stimulation (Pandey and Sharma, 2011). Therefore, incorporating potassium into biomaterials and achieving controlled release may have great potential to promote nerve regeneration in bone-fracture areas.

Herein, we constructed a titanium dioxide (TiO_2_) nanotube array by an anodic oxidation process and assembled a layer of K_2_Ti_6_O_13_-TiO_2_ nanorods on the channel surface of the TiO_2_ nanotubes. The prepared K_2_Ti_6_O_13_-TiO_2_ hybrid nanotube arrays was worked as a controlled potassium-release platform to explore the neurogenesis performance. The synergistic effect of potassium stimulation and nanotopography can promote the differentiation of NSCs into nerve cells and simultaneously enhance the osteogenic differentiation of mesenchymal stem cells. This multifunctional biomaterial is a feasible solution for the repair of bone defects, which has promising potential for future applications in the formation of a fully functional interface in bone repair.

## Materials and Methods

### Chemicals

Ti foil was purchased from Xinji Metal Materials Co., Ltd., (China). Ammonium fluoride, potassium hydroxide, ethylene glycol, ethanol, and sodium dodecyl sulfate (SDS) were obtained from Sinopharm Chemical Reagent Co., Ltd. Bovine serum albumin was obtained from Sigma-Aldrich. Tissue culture plate was purchased from Biosharp. Phalloidin labeled with Alexa Fluor 555, 4′,6-diamidino-2-phenylindole (DAPI), and Trizol reagent were obtained from Invitrogen. A reverse-transcription kit and SYBR Green^®^ Premix Pro Taq™ HS qPCR Kit II were purchased from Accurate Biotechnology (Hunan) Co., Ltd., and β-NGF, BMP-2, and VEGF were purchased from Peprotech.

### Preparation of Titanium Dioxide Nanotube Array

Ti foils were ground using abrasive paper and ultrasonically cleaned in acetone, ethanol, and deionized water. The TiO_2_ nanotube array was fabricated by two anodization steps using a two-electrode configuration with a Ti plate as the anode and another Ti plate as the cathode, similar to those described previously (Gong et al., 2017). Briefly, the first step of anodization was performed at 50 V for 30 min; then, the Ti foil was ultrasonically cleaned in hydrogen peroxide for 10 min to remove the nanotubes. The second step of anodization was performed at 30 V for 30 min to obtain a double-layered TiO_2_ array. The electrolyte consisted of ethylene glycol solution with 0.3 wt% NH_4_F and 2 vol% deionized water. During the two anodization steps, a magnetic stirrer was used to maintain the uniformity and stability of the electrolyte solution. After anodization, the sample was gently washed with deionized water and dried in air. Finally, the samples were annealed at 450°C for 2 h in air.

### Preparation of Potassium Titanate Nanorod-Decorated Titanium Oxide Hybrid Nanorod Array

The as-prepared TiO_2_ nanotube arrays were placed in 50 ml of 5 M KOH solution in a Teflon-lined autoclave and heated at 150°C for 24 h to obtain the K_2_Ti_6_O_13_-TiO_2_ arrays. The sample was then washed with distilled water and ethanol for several times and dried in air.

### Characterization

The morphology was observed using an S-4800 (Hitachi, Japan) scanning electron microscope (SEM). X-ray diffractograms (XRD) were recorded on a Bruker D8 Advance powder diffractometer equipped with a Cu Kα sealed tube to analyze the elemental composition. FT-IR spectra were recorded using an infrared spectrophotometer (Nicolet Nexus 670, Thermo Fisher Scientific, Inc.). Raman spectra were recorded by Raman spectroscopy (LabRAM HR Evolution). XPS was conducted on an AXIS SUPRA (Shimadzu, Japan) using 200-W monochromatic Al *Kα* radiation. Inductively coupled plasma (ICP) analysis was conducted using a Thermo iCAP 7200 ICP-OES instrument.

### Mesenchymal Stem Cells Culture and Differentiation

For the MSC proliferation culture, MSCs were cultured in α-MEM supplemented with 10% FBS and 1% penicillin/streptomycin. The cells were maintained in humidified air with 5% CO_2_ at 37°C, and the culture medium was changed every 2 days.

For the MSC differentiation culture, MSCs were maintained in osteogenic differentiation medium (basal medium plus 10 × 10^−9^ M dexamethasone, 10 × 10^−3^ M β-glycerophosphate, and 50 mg mL^−1^ L-ascorbic acid), and the medium was changed every 2 days.

### Neural Stem Cells Culture and Differentiation

For the NSC proliferation culture, NSCs were cultured in neurobasal medium supplemented with 2% B-27 supplement, 1% glutaMAX™-1, 20 ng/ml EGF, 20 ng/ml bFGF, and 1% penicillin/streptomycin, and maintained in humidified air with 5% CO_2_ at 37°C.

For the NSC differentiation culture, NSCs were maintained in neurobasal medium supplemented with 2% B-27 supplement, 1% glutaMAX™−1, 1% FBS, and 1% penicillin/streptomycin in culture dishes pre-coated with poly-L-lysine (10 μg/ml).

### Cell Proliferation and Live/Dead Staining *in vitro*

To test the cell proliferation ability, we seeded the cells in 96-well culture plates at a density of 4 × 10^3^ per well, or on TiO_2_ nanotube arrays or K_2_Ti_6_O_13_-TiO_2_ hybrid nanorod arrays. They were then cultured for several days and detected using a Cell Counting Kit-8 (CCK-8). For live and dead cell staining, cells were seeded in 48-well plates at 1 × 10^4^ per well or on TiO_2_ nanotube or K_2_Ti_6_O_13_-TiO_2_ hybrid nanorod arrays for 2 days. The cells were washed and treated with 200 μl of serum-free medium containing 1 μM of calcein AM (APExBIO) and 4 μM of PI. After incubation for 30 min at 37°C, the cells were washed with phosphate buffer solution (PBS) for three times and observed under a fluorescence microscope (Olympus).

### Real-Time Quantitative PCR

For the RT-qPCR assay, NSCs and MSCs were seeded on different substrates (24-well culture plates, TiO_2_ nanotube arrays, and K_2_Ti_6_O_13_-TiO_2_ hybrid nanorod arrays) at a density of 4 × 10^4^ cells per well and cultured for 7 and 14 days, respectively. Trizol reagent was used to extract the total RNA, and the concentration and purity were determined using a Q-5000 spectrophotometer (Quawell, Q-5000, CA, United States) at 260/280 nm. DNA-free total RNA (500 ng) was reverse-transcribed according to the manufacturer’s instructions. Finally, the signals were measured using a 7500 Real-Time PCR system (Applied Biosystems, Germany) to analyze the expression of mouse neural-related markers Nestin, Tuj1, MAP2, and GFAP, and rat osteogenic-related markers Runx2, OPN, and OCN (primer sequences are provided in Supplementary Tables S1, S2). β-actin served as an internal reference for RNA quantification of NSCs, and glyceraldehyde-3-phosphate dehydrogenase (GAPDH) was used as an internal reference for RNA quantification of MSCs. All target genes were expressed as mean ± SD (*n* = 3 for each group).

### Scanning Electron Microscope Observation of Neural Stem Cells Samples by Alcohol Gradient Dehydration

NSCs were seeded on K_2_Ti_6_O_13_-TiO_2_ hybrid nanorod arrays at a density of 5 × 10^3^ cells per well. After culturing on substrates for 7 days, NSCs were fixed with 2.5% glutaraldehyde solution in PBS for 30 min at 25°C. The samples were then washed three times with PBS and dehydrated using an alcohol gradient (30, 50, 70, 80, 90, 95, 98, and 100%). The samples were then lyophilized at −80°C for 12 h and finally sprayed with Au at a current of 20 μA and observed under SEM.

### Immunofluorescence Staining

Cells were seeded on different substrates (24-well culture plates, TiO_2_ nanotube arrays, and K_2_Ti_6_O_13_-TiO_2_ hybrid nanorod arrays) at a density of 4 × 10^3^ per well. After culturing on different substrates for 7 and 14 days, NSCs and MSCs were washed with PBS for three times and fixed with 4% paraformaldehyde at room temperature for 20 min. The samples were then permeabilized for 10 min with 0.1% Triton X-100 and blocked for 30 min at room temperature with 1% bovine serum albumin. After blocking, the cells were incubated overnight at 4°C with primary antibodies in 1% bovine serum albumin. The secondary antibody was used for staining for 1 h at room temperature. After washing three times with PBS, the cells were stained with DAPI for 5 min. Finally, the samples were observed under different excitation wavelengths using a Confocal laser scanning microscope (CLSM).

### Western Blot Assay

The western blot assay was performed as described previously (Yu et al., 2016). NSCs and MSCs were prepared using RIPA buffer. The lysate protein concentrations were determined using a BCA protein assay kit (Beyotime Biotechnology, Nanjing, China). The protein samples were denatured in Laemmli buffer at 100°C for 10 min before electrophoresis, and were then subjected to 10% SDS-PAGE before being electrotransferred to polyvinylidene difluoride membranes with a standard transfer solution. After blocking with 10% nonfat milk, membranes were incubated with primary antibodies against β-tubulin III (1:1,000; Abcam, Cambridge, MA), MAP2 (1:1,000; Abcam, Cambridge, MA), Runx2 (1:1,000; Abcam, Cambridge, MA), BMP-2 (1:2000; ProteinTech Group), and OPN (1:2000; ProteinTech Group), and GAPDH antibody as a control (1:5,000; ProteinTech Group). Proteins were visualized by chemiluminescence using an ECL kit (Share-Bio, Jinan, China).

### Calcium Imaging

Cells were seeded on K_2_Ti_6_O_13_-TiO_2_ hybrid nanorod arrays at a density of 4 × 10^3^ per well. After culturing for 7 days, cells were washed with PBS and labeled with Fluo-4 AM (S1060, Beyotime) to visualize intracellular calcium ions distribution. CLSM was employed to image labeled cells. For calcium spark experiments, cells were stimulated with neurotransmitters and images were taken at a frequency of 1 Hz.

### Statistical Analysis

Statistical analysis consisted of a one-way ANOVA in GraphPad Instant software (GraphPad Software), followed by Duncan’s multiple range test. Data are reported as the mean ± standard deviation, and statistical significance was accepted at *p* < .05.

## Results and Discussion

### Material Characterization

**FIGURE 1:**
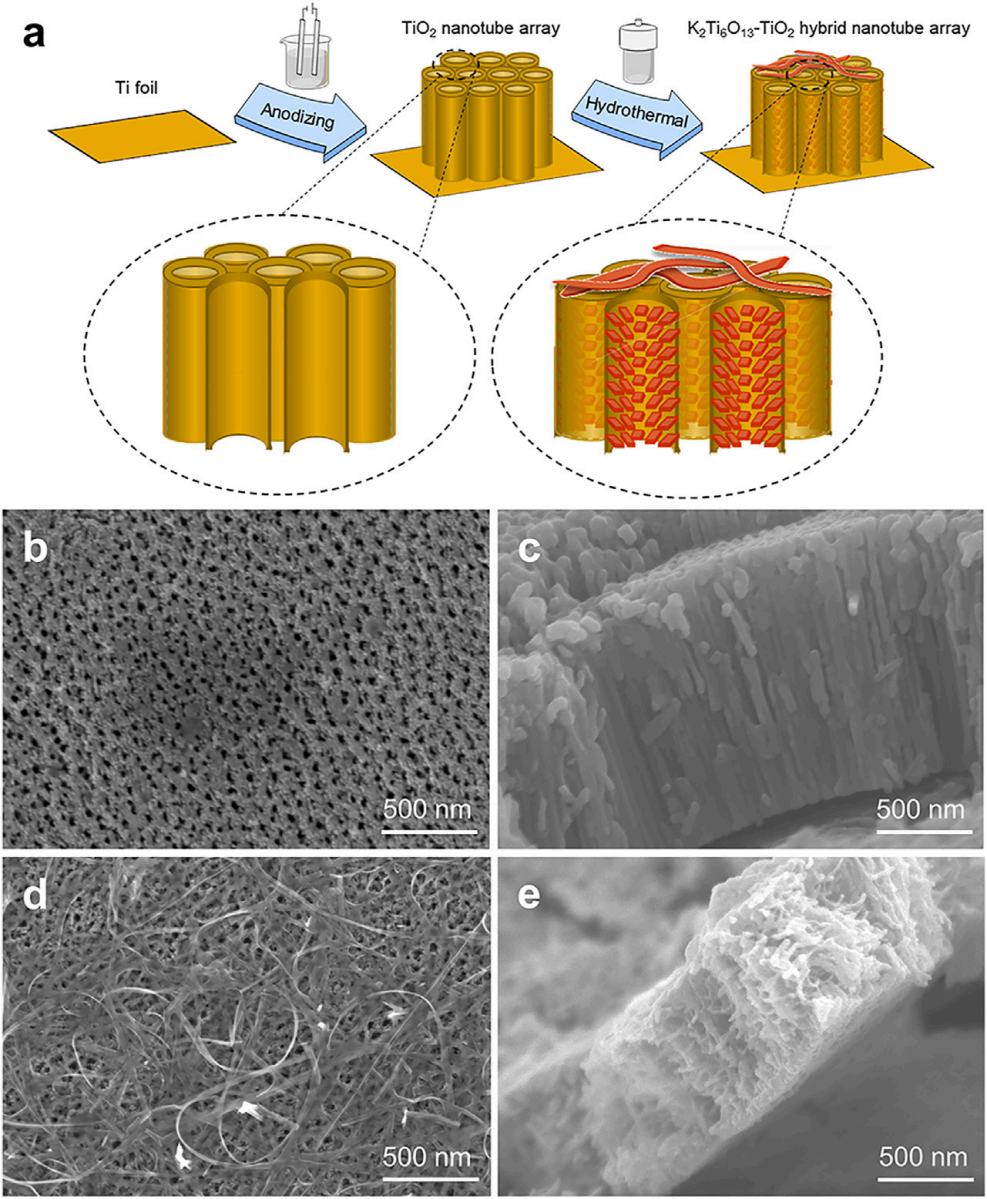
Schematic illustration of TiO_2_ and K_2_Ti_6_O_13_-TiO_2_ hybrid nanotube arrays. **(A)** Schematic illustration of the K_2_Ti_6_O_13_-TiO_2_ hybrid nanotube array preparation process. SEM images of the **(B)** Top-down view SEM images of TiO_2_ nanotube array, **(C)** cross-sectional SEM image of TiO_2_ nanotube array, **(D)** Top-down view SEM images of K_2_Ti_6_O_13_-TiO_2_ hybrid nanotube array, and **(E)** cross-sectional SEM image of K_2_Ti_6_O_13_-TiO_2_ hybrid nanotube array.

Schematic illustrations of the TiO_2_ and K_2_Ti_6_O_13_-TiO_2_ hybrid nanotube arrays were shown in Figure 1A. TiO_2_ nanotube arrays were electrochemically synthesized *in situ* on titanium foil by a self-assembled anodic oxidation method (Hou et al., 2021). Then, a hydrothermal treatment was applied in a 5 M KOH solution to assemble a potassium titanate nanorod array on the surface of the channel of TiO_2_ nanotubes and formed the K_2_Ti_6_O_13_-TiO_2_ hybrid nanotube array. Figures 1B–E showed the morphology of the TiO_2_ nanotube arrays before and after the hydrothermal treatment. As shown in Figures 1B,C, the TiO_2_ nanotube arrays were packed tightly, with a height of approximately 1.5 μm, and the TiO_2_ nanotube array layer was tightly connected to the titanium substrate. The diameter of the TiO_2_ nanotube was approximately 70–80 nm. Figures 1D,E showed the SEM images of K_2_Ti_6_O_13_-TiO_2_ hybrid nanotube array. Obviously, a few belt-like nanostructures were found on the top of the nanotube array (Figure 1D). More interestingly, the inner morphology of the K_2_Ti_6_O_13_-TiO_2_ nanotubes is different from that of TiO_2_, and some nanorods were uniformly distributed on the wall of the K_2_Ti_6_O_13_-TiO_2_ nanotubes. XRD was employed to characterize the crystalline structures of the resultant samples. All the diffraction peaks of the sample could be easily indexed to anatase TiO_2_ and Ti, corresponding to JCPD cards #21-1272 and #44-1294, respectively. After hydrothermal treatment, a very broad peak could be found at approximately 28°, which is the peak of K_2_Ti_6_O_13_ (Janes and Knightley, 2004) (Supplementary Figure S1). Combined with the SEM observations, the phase with rod-like nanostructures on the channel surface and belt-like nanostructures in Figures 1D,E should belong to the crystal K_2_Ti_6_O_13_.

**FIGURE 2:**
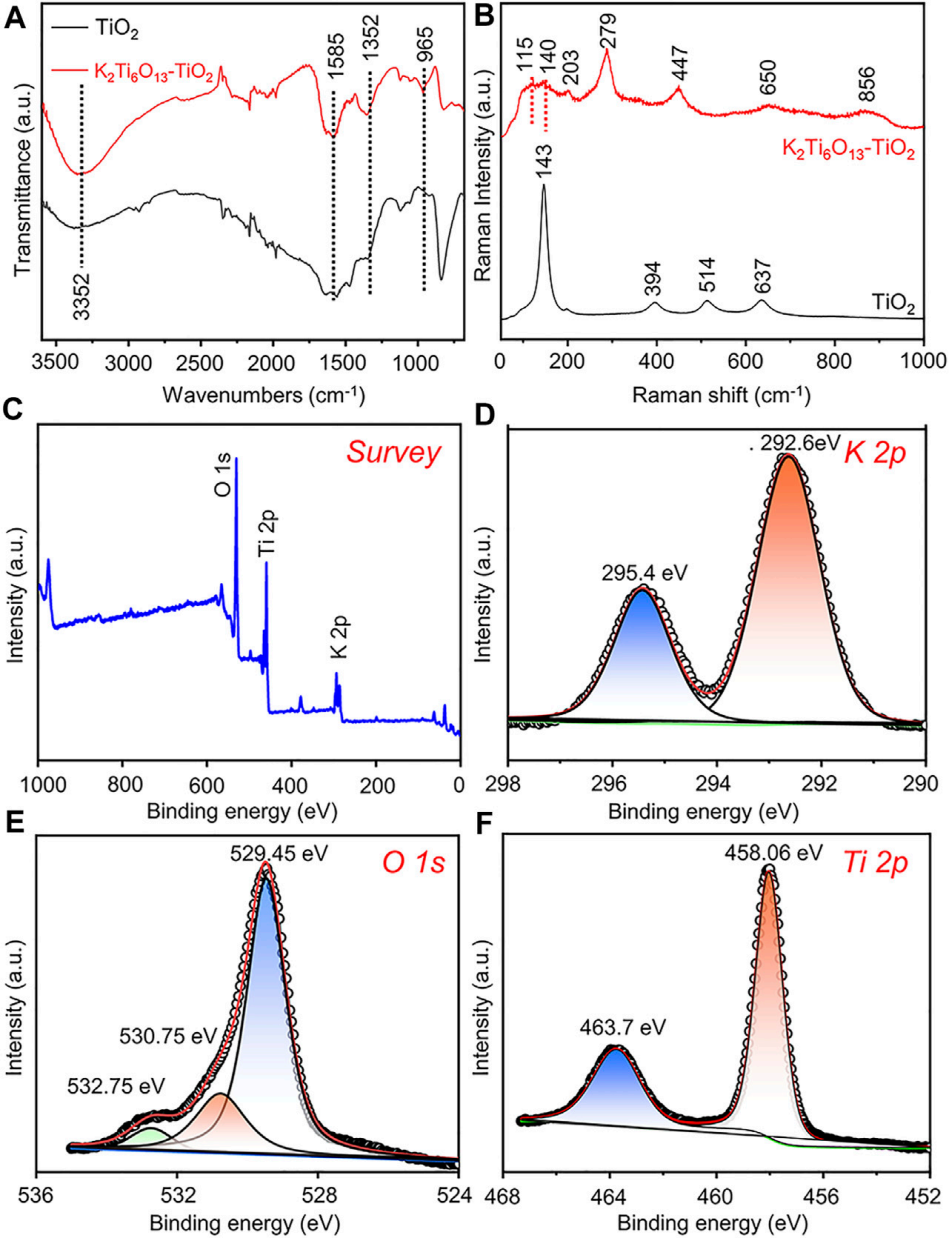
Characterization of K_2_Ti_6_O_13_-TiO_2_ hybrid nanorod arrays and TiO_2_ nanotube arrays. **(A)** FTIR analysis of K_2_Ti_6_O_13_-TiO_2_ hybrid nanorod arrays and TiO_2_ nanotube arrays. **(B)** Raman spectra of K_2_Ti_6_O_13_-TiO_2_ hybrid nanorod arrays and TiO_2_ nanotube arrays. XPS spectra of K_2_Ti_6_O_13_-TiO_2_ hybrid nanorod arrays: **(C)** survey, **(D)** K 2p, **(E)** O 1s, and **(F)** Ti 2p spectra.

To further verify the formation of the K_2_Ti_6_O_13_-TiO_2_ hybrid nanorod arrays, Fourier transform-infrared (FT-IR) spectroscopy and Raman spectroscopy were also conducted. In Figure 2A, the characteristic absorption peak of TiO_2_ could be observed at 800 cm^−1^, which was assigned to a combination of Ti-O-Ti vibration in crystalline TiO_2_ (Sim et al., 2014). After the hydrothermal process, the characteristic absorption peak at 800 cm^−1^ disappeared, and the K_2_Ti_6_O_13_-TiO_2_ hybrid nanotube array exhibited several new absorption peaks in the range of 1,500–500 cm^−1^. The adsorption peak at 935 cm^−1^ was attributed to the absorption band of the TiO_6_ octahedral framework of the K_2_Ti_6_O_13_-TiO_2_ hybrid nanotube array (Sasani Ghamsari et al., 2012). The peaks at 1,585 and 3,352 cm^−1^ were assigned to the absorption of OH^−1^ (Masaki et al., 2002). The Raman spectra of the TiO_2_ and K_2_Ti_6_O_13_-TiO_2_ hybrid nanotube arrays were presented in Figure 2B. Pure TiO_2_ nanotube arrays exhibited Raman bands at 144, 394, 514, and 634 cm^−1^, which were attributed to the typical anatase. After hydrothermal treatment, the K_2_Ti_6_O_13_-TiO_2_ hybrid nanotube array exhibited a series of new Raman peaks at 115, 140, 203, 279, 447, 650, and 850 cm^−1^, which were attributed to the formation of new potassium titanate compound K_2_Ti_6_O_13_, consistent with a previous report (Bamberger et al., 1990). X-ray photoelectron spectroscopy (XPS) (Figures 2C–F) was performed on the K_2_Ti_6_O_13_-TiO_2_ hybrid nanorod arrays to further confirm the existence of potassium in the samples. The K 2p spectra were also detected from the K_2_Ti_6_O_13_-TiO_2_ hybrid nanorod arrays (Figure 2D), and the two strong peaks located at 295.4 and 292.6 eV could be ascribed to K 2p_1/2_ and K 2p_3/2_, respectively (Liu et al., 2019). The asymmetric O 1s signal (Figure 2E) indicated that several oxygen species coexisted on the surface of the K_2_Ti_6_O_13_-TiO_2_ hybrid nanorod arrays. After deconvolution, the O 1s XPS spectrum could be fitted with three peaks corresponding to K-O bonds (529.45 eV), Ti-O bonds (530.75 eV), and surface hydroxyl oxygen (532.75 eV) (Nassoko et al., 2012; Zhang et al., 2018). In addition to the peaks of K and O, Figure 2F showed high-resolution XPS scans of the Ti 2p peaks. Two XPS peak at 458.06 and 463.7 eV could be assigned to Ti^4+^ 2p_3/2_ and Ti^4+^ 2p_1/2_ (Pang et al., 2021). These results suggested that Ti was mainly present as TiO_2_ on the surface of the K_2_Ti_6_O_13_-TiO_2_ hybrid nanorod arrays. The above experimental results demonstrated the successful synthesis of K_2_Ti_6_O_13_-TiO_2_ hybrid nanorod array.

### Cytocompatibility of the Titanium Dioxide and Potassium Titanate Nanorod-Decorated Titanium Oxide Hybrid Nanorod Arrays

NSCs and MSCs were selected to assess the cytocompatibility and differentiation-inductive ability of different substrates. Before the cytocompatibility and differentiation experiments, Nestin immunofluorescence staining was used to assess the quality of NSCs (Supplementary Figure S2). Almost all of the NSCs on the culture plate were nestin-positive, indicating the high multi-differential potential of the isolated NSCs.

**FIGURE 3:**
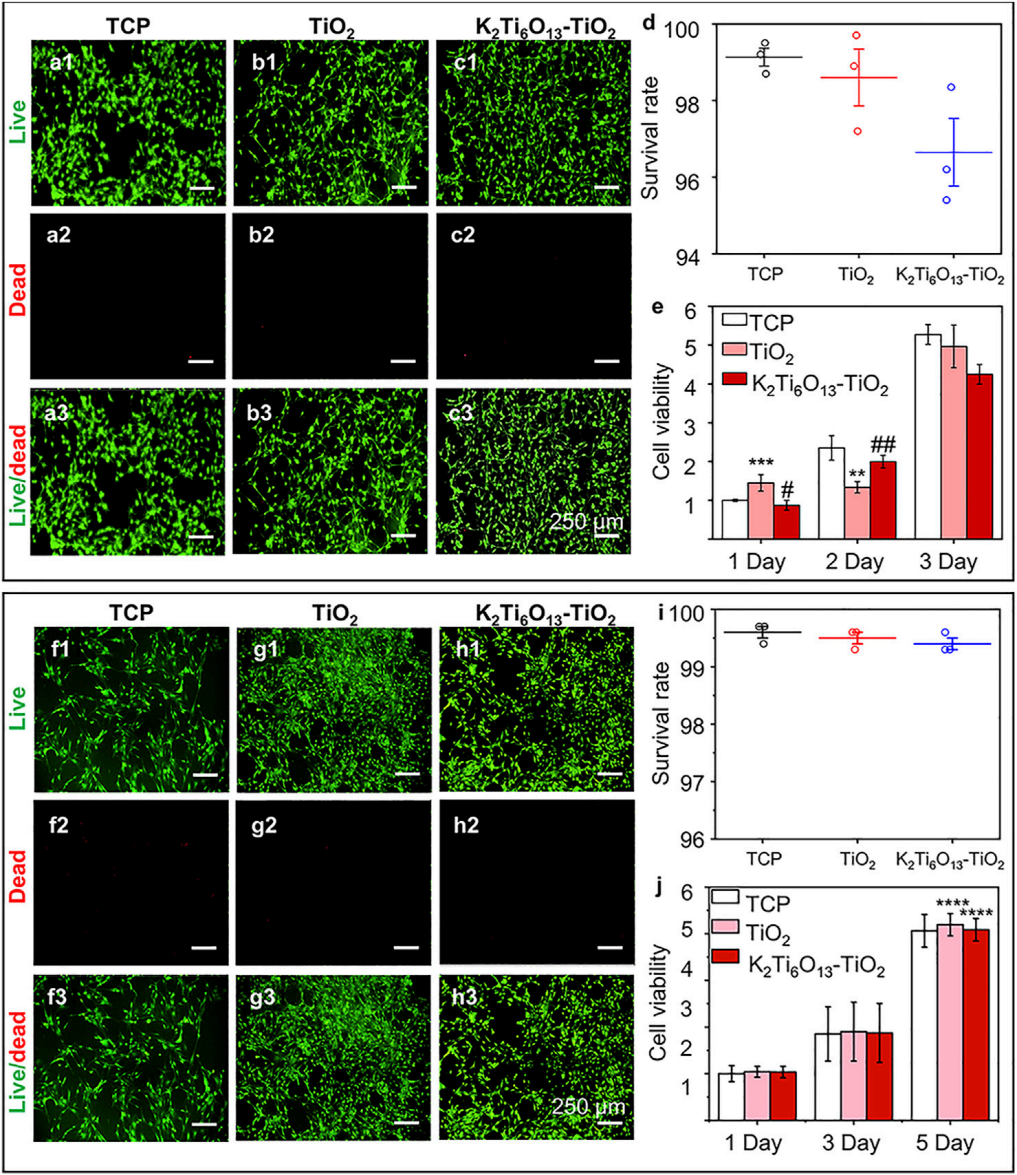
Cell viability of NSCs and MSCs cultured on different substrates., **(A–C)** Live/dead cellular staining images of NSCs seeded on the **(A)** tissue culture plate, **(B)** TiO_2_ nanotube array, and **(C)** K_2_Ti_6_O_13_-TiO_2_ hybrid nanorod array for 48 h, respectively. Live cells were stained green and dead cells were stained red. **(D)** Survival rate of NSCs on three substrates for 48 h. **(E)** Cell viability of cultured NSCs at 1, 2, and 3 d **(F–H)** Live/dead cellular staining images of MSCs seeded on the **(F)** tissue culture plate, **(G)** TiO_2_ nanotube array, and **(H)** K_2_Ti_6_O_13_-TiO_2_ hybrid nanorod array for 48 h. **(I)** Survival rate of MSCs on three substrates for 48 h., **(J)** Cell viability of cultured MSCs at 1, 3, and 5 days. Data represent the mean ± standard deviation (*n* = 3). ^*^ *p* < .05, ^**^ *p* < .01, ^***^ *p* < .001 compared with TCP; ^#^ *p* < .05, ^###^ *p* < .001 compared with TiO_2_ array. Scale bars in **(A–C)** and **(F–H)** are 250 μm.

To assess the cytocompatibility of the samples, NSCs and MSCs were cultured on tissue culture plates (TCP), TiO_2_ nanotube arrays, and K_2_Ti_6_O_13_-TiO_2_ hybrid nanorod arrays for 2 days, and stained with a Live/Dead kit to characterize the cell viability on different materials. The Live/Dead assessment results of NSCs and MSCs cultured on these materials were shown in Figures 3A,B,C,F,G,H, respectively. The live cells were stained green by Calcein AM, while dead cells were stained red with propidium iodide (PI). For NSCs, the number of living NSCs cultured on K_2_Ti_6_O_13_-TiO_2_ hybrid nanorod arrays was similar to that of the TiO_2_ nanotube arrays, while the dead cell population on the TiO_2_ nanotube arrays was slightly less than that on the K_2_Ti_6_O_13_-TiO_2_ hybrid nanorod arrays. The same tendency was observed in the MSC group. The population of living MSCs seeded on K_2_Ti_6_O_13_-TiO_2_ hybrid nanorod arrays was similar to that of the TCP group, and the number of living MSCs cultured on TiO_2_ nanotube arrays was slightly higher than that in the TCP group. Moreover, dead cells in both the TiO_2_ nanotube and K_2_Ti_6_O_13_-TiO_2_ hybrid nanorod arrays groups decreased when compared with that of TCP group, indicating the good cytocompatibility of these scaffolds.

The survival rates of NSCs and MSCs cultured with different nanotube arrays were determined using ImageJ, and the results are shown in Figures 3D,I, respectively. For NSCs, the ratio of live cells for all samples exceeded 95%, while the survival rates of all three groups exceeded 99% for the MSCs, suggesting that NSCs and MSCs could survive on the three substrates; that is, TiO_2_ and K_2_Ti_6_O_13_-TiO_2_ hybrid nanorod arrays had low cytotoxicity during cell cultivation.

To quantitatively evaluate cell viability, the abovementioned model cells were cultured on TiO_2_ and K_2_Ti_6_O_13_-TiO_2_ hybrid nanorod arrays for several days, and CCK-8 tests were conducted to estimate the cell proliferation ability (Figures 3E,J, respectively). The number of cells increased in a time-dependent manner over 3 days. NSCs cultured on the three substrates exhibited similar proliferation rates at 1, 2, and 3 days. However, the cell population on the K_2_Ti_6_O_13_-TiO_2_ hybrid nanorod arrays was slightly lower than that of other groups on day 3, which may be attributed to more NSCs cultured on K_2_Ti_6_O_13_-TiO_2_ hybrid nanorod arrays tended to differentiate. This phenomenon is in accord with the results in previous works, in which a decrease in proliferation is prior to differentiation (Liu et al., 2013; Malec et al., 2016). For MSCs, the viability of all three substrates increased with increasing culture time, and the viability of the K_2_Ti_6_O_13_-TiO_2_ hybrid nanorod arrays was equal to that of the other two substrates. These results demonstrate that the as-prepared K_2_Ti_6_O_13_-TiO_2_ hybrid nanorod arrays possess good cytocompatibility, and are adequate for further research.

### Promotion Effect of Potassium Titanate Nanorod-Decorated Titanium Oxide Hybrid Nanorod Arrays on Osteogenic Differentiation of MSCs

**FIGURE 4:**
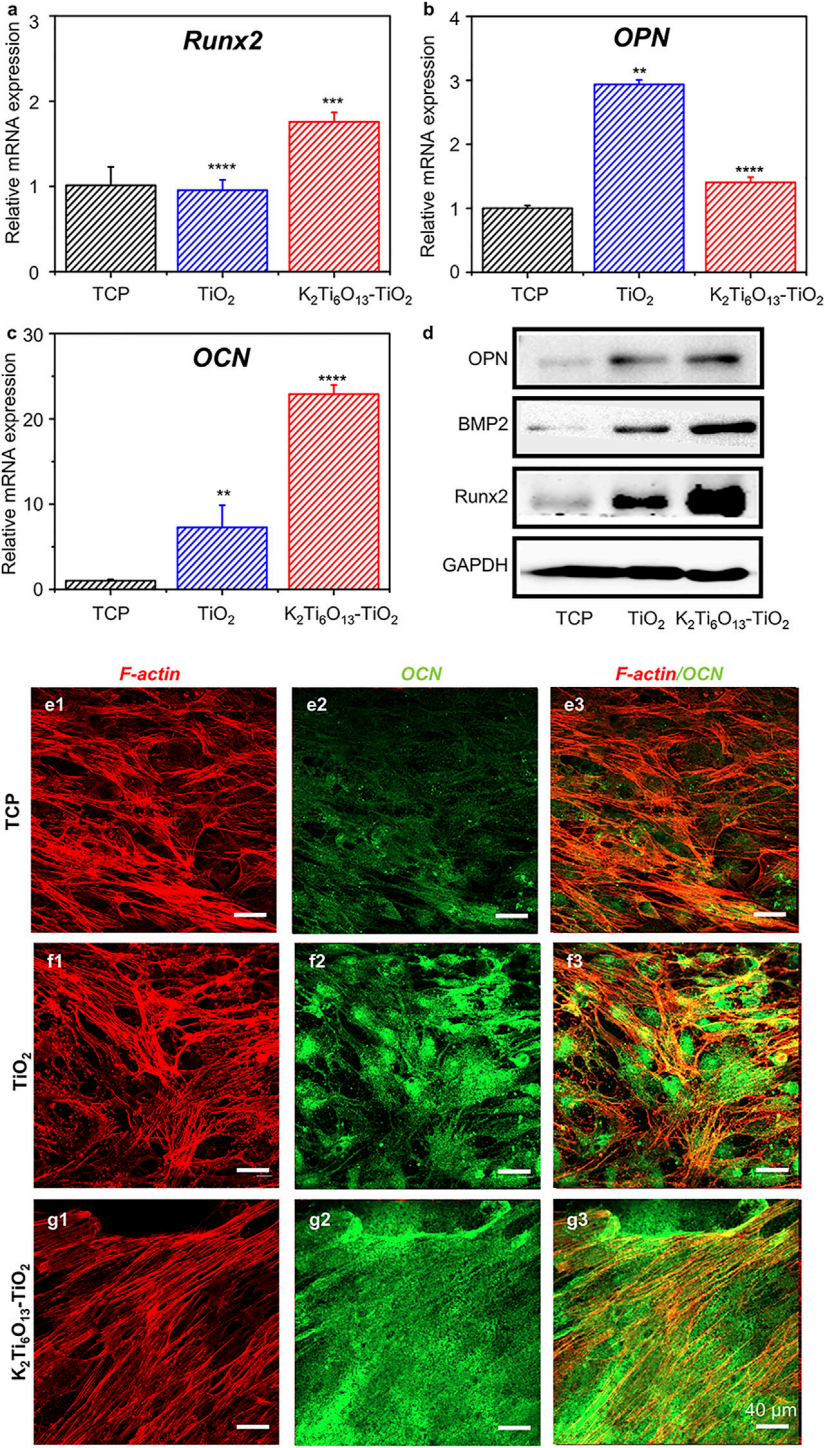
Gene and protein expression of the osteogenic differentiation of MSCs on TiO_2_ nanotube and K_2_Ti_6_O_13_-TiO_2_ hybrid nanorod arrays for 14 days. Osteogenic-related mRNA expressions of **(A)** Runx2, **(B)** OPN, and **(C)** OCN. **(D)** Western blot images of osteogenic marker, OPN, BMP2., Runx2, **(E–G)** Immunofluorescence cellular staining images of the cytoskeleton (red) and OCN (green). Data represent the mean ± standard deviation (*n* = 3). ^***^ *p* < .001 compared with TCP. Scale bars in **(E–G)** are 40 μm.

First, we assessed the osteogenic-induced function of K_2_Ti_6_O_13_-TiO_2_ hybrid nanorod arrays. MSCs were cultured on TiO_2_ and K_2_Ti_6_O_13_-TiO_2_ hybrid nanorod arrays in osteogenic induction media. Tissue culture plates were used as positive controls. After 14 days of culture, the real-time quantitative polymerase chain reaction (RT-qPCR) was used to evaluate the expression of osteogenesis-related genes in cultured cells. Runt-related transcription factor 2 (Runx2) is one of the most important transcription factors during osteogenic differentiation (Komori, 2019). As shown in Figure 4A, the relative mRNA level of Runx2 in the group seeded on K_2_Ti_6_O_13_-TiO_2_ hybrid nanorod arrays was up-regulated to 1.76 folds compared with that of the control group, while the expression level of Runx2 in the TiO_2_ group was not significantly different to that in the control group. Osteocalcin (OCN) is the most accurate marker of osteogenesis (Komori, 2020). The relative OPN mRNA expression level of TiO_2_ was 2.93 folds, higher than that of the K_2_Ti_6_O_13_-TiO_2_ hybrid nanorod array group (1.4 folds) (Figure 4B). The expression of the osteogenic differentiation marker OPN peaked at 7 days, which could be attributed to the relative late-stage osteogenic differentiation in the K_2_Ti_6_O_13_-TiO_2_ hybrid nanorod array group (Tang et al., 2017). Figure 4C showed a dramatic improvement in the relative OCN mRNA expression in the group of K_2_Ti_6_O_13_-TiO_2_ hybrid nanorod arrays. In contrast with the TCP group, the relative mRNA expression level of the K_2_Ti_6_O_13_-TiO_2_ hybrid nanorod arrays was enhanced by 22.2 folds, and the relative OCN mRNA expression level of TiO_2_ increased to 7.2 folds.

To further confirm the promotion effect of K_2_Ti_6_O_13_-TiO_2_ hybrid nanorod arrays on the osteogenic differentiation of MSCs, western blot assay was employed to assess osteogenic differentiation at the protein level. MSCs were seeded on TiO_2_ and K_2_Ti_6_O_13_-TiO_2_ hybrid nanorod arrays and cultured for 14 days in osteogenic induction media. As shown in Figure 4D, the expression of bone morphogenetic protein-2(BMP-2) and Runx2 increased in the following order: K_2_Ti_6_O_13_-TiO_2_ hybrid nanorod arrays > TiO_2_ nanotube arrays > TCP.

Immunofluorescence staining was also conducted to visualize the expression of OCN using a confocal microscope. The cytoskeleton was marked red, and the osteogenic-related protein OCN was marked green. After culturing for 14 days, the cell morphology exhibited no obvious difference in all groups; however, the expression of OCN in the K_2_Ti_6_O_13_-TiO_2_ hybrid nanorod array group was significantly higher than that in the TCPs and TiO_2_ groups. The K_2_Ti_6_O_13_-TiO_2_ hybrid nanorod array group exhibited the highest relative OCN fluorescence intensity, which was 0.46-fold higher than that of the TCP group (Supplementary Figure S3). However, the relative fluorescence intensity expressed in the TiO_2_ group was only 0.35 folds higher than that of the TCP group.

These data indicate that the K_2_Ti_6_O_13_-TiO_2_ hybrid nanorod arrays possess a prominent ability to promote the osteogenic differentiation of MSCs, which is of vital significance for constructing a fully functional interface between a titanium implant and the surrounding native bone tissue.

### Promotion Effect of Potassium Titanate Nanorod-Decorated Titanium Oxide Hybrid Nanorod Arrays on Neural Differentiation of Neural Stem Cells

**FIGURE 5:**
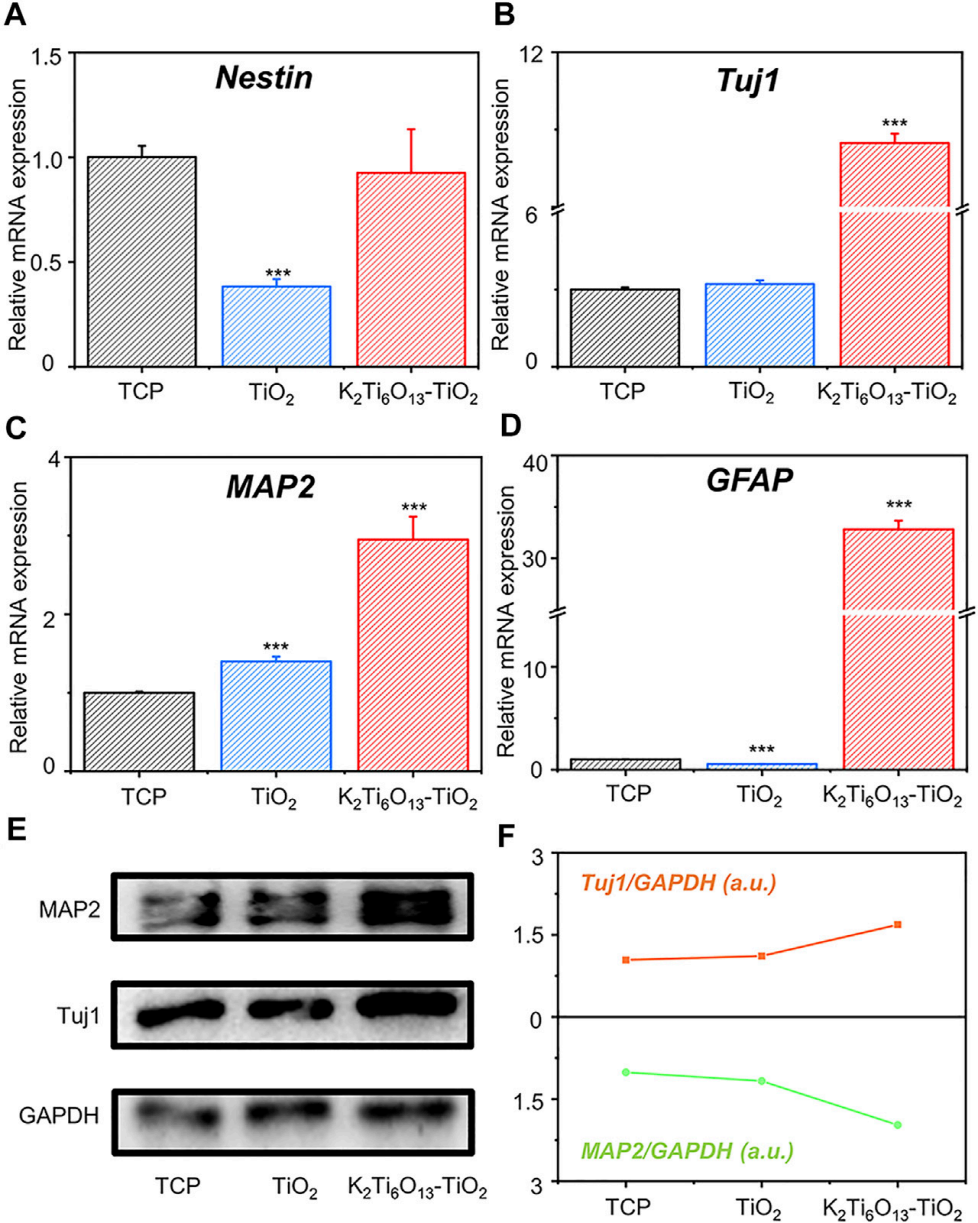
Gene and protein expression of the neural differentiation of NSCs on TiO_2_ nanotube and K_2_Ti_6_O_13_-TiO_2_ hybrid nanorod arrays for 7 days. Relative mRNA expression of **(A)** Stemness gene Nestin, **(B)** Neuromarker Tuj1, **(C)** Neuromarker MAP2, and **(D)** Glial cells marker GFAP. **(E)** Western blot image and **(F)** quantitative analysis of neural markers Tuj1 and MAP2. Data represent the mean ± standard deviation (*n* = 3). ^***^ *p* < .001 compared with TCP.

To explore whether the K_2_Ti_6_O_13_-TiO_2_ hybrid nanorod arrays can accelerate the neural differentiation of NSCs, NSCs were seeded on TiO_2_ and K_2_Ti_6_O_13_-TiO_2_ hybrid nanorod arrays. TCPs were used as the blank control group. NSCs were cultured on different samples for 7 days, and RT-qPCR was performed to analyze the expression of neurospecific genes in the cultured cells. Four typical specific markers were selected to evaluate neurogenic differentiation, including Nestin, β-tubulin-III (Tuj1), microtubule-associated protein 2 (MAP2), and Glial fibrillary acidic protein (GFAP). Figure 5A showed the relative mRNA expression levels of Nestin normalized to those of the TCPs. The cells cultured on TiO_2_ nanotube arrays and K_2_Ti_6_O_13_-TiO_2_ hybrid nanorod arrays exhibited relatively lower mRNA expression levels of nestin than those in the control group, suggesting that the stemness of NSCs seeded on these substrates was depressed. As shown in Figure 5B, the relative mRNA expression level of Tuj1 in the group cultured on K_2_Ti_6_O_13_-TiO_2_ hybrid nanorod arrays was up-regulated to 10.8 folds from that of those cultured on TCPs. There was no significant difference between the group cultured on TiO_2_ nanotube arrays and that cultured on TCPs. A similar trend was observed for the expression of MAP2. As shown in Figure 5C, the expression of MAP2 in the cells on the K_2_Ti_6_O_13_-TiO_2_ hybrid nanorod arrays was 1.9 folds higher than that on the TCPs. Tuj1 and MAP2, which significantly affect neurogenesis, are the two most important types of representation forms during the neurogenic differentiation period (Liu et al., 2021). The above experimental results show that NSCs cultured on K_2_Ti_6_O_13_-TiO_2_ hybrid nanorod arrays present higher neuron-related gene expression levels than those on TCPs or TiO_2_ arrays, indicating that the K_2_Ti_6_O_13_-TiO_2_ hybrid nanorod arrays possess excellent acceleration functions for the neurogenic differentiation of NSCs. GFAP expression was upregulated by more than 30-fold in the K_2_Ti_6_O_13_-TiO_2_ hybrid nanorod arrays compared with that on TCPs. As shown in Figure 5D, the relative mRNA expression level of GFAP decreased was 0.55 folds lower than that on TCPs. Astrocytes exhibited high resting K^+^ conductance, which facilitated the uptake and buffering of neuronally released K^+^ (Durand et al., 2010; Bellardita et al., 2012). This significant gene expression enhancement could be attributed to the adaptation of cells to the elevated K^+^ ion concentration in the extracellular matrix.

To further confirm the acceleration effect of K_2_Ti_6_O_13_-TiO_2_ hybrid nanorod arrays on neural stem cell differentiation at the protein level, western blotting and immunofluorescence staining were conducted on the NSCs cultured on the different samples. After 7 days of culturing, the western blot analysis (Figure 5E) showed that the protein expression of the neural-specific markers Tuj1 and MAP2 on K_2_Ti_6_O_13_-TiO_2_ hybrid nanorod arrays was higher than that on the TiO_2_ nanotube array and TCP. The expression of Tuj1 in the TiO_2_ and K_2_Ti_6_O_13_-TiO_2_ hybrid nanorod array groups increased to 1.11 and 1.68 folds. Additionally, the expression of MAP2 exhibited the same trends in the TiO_2_ and K_2_Ti_6_O_13_-TiO_2_ hybrid nanorod array groups, increasing to 1.17 and 1.68 folds, respectively (Figure 5F).

**FIGURE 6:**
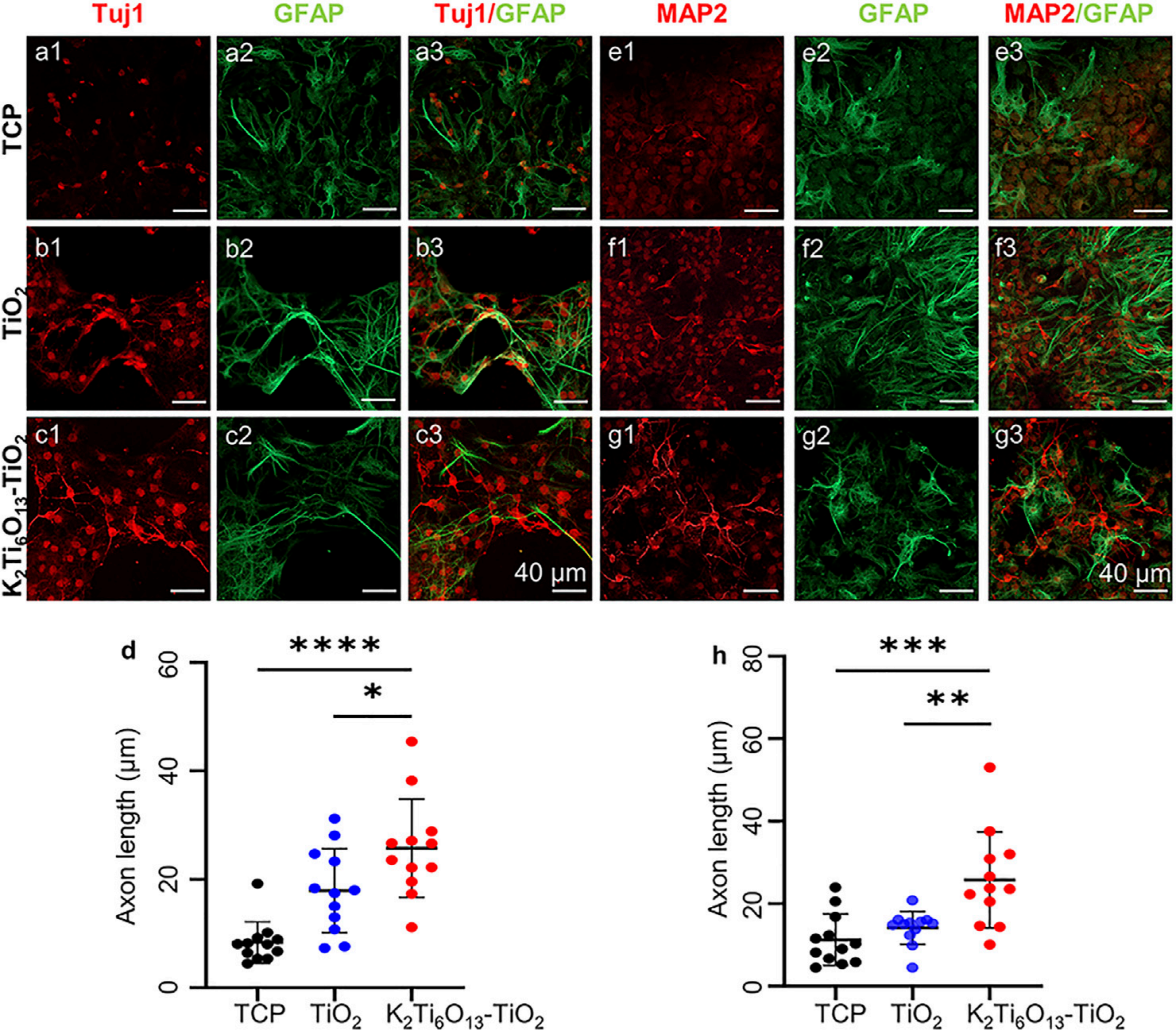
Immunofluorescence cellular staining images of neural markers., **(A–C)** Immunofluorescence cellular staining images of Tuj1 (red), and GFAP (green) for 7 days **(D)** Axon length statistics of Tuj1-positive neurons on TCP, TiO_2_ nanotube array, and K_2_Ti_6_O_13_-TiO_2_ hybrid nanorod array. **(E–G)** Immunofluorescence cellular staining images of MAP2 (red) and GFAP (green) for 7 days. **(H)** Axon length statistics of MAP2-positive neurons on TCP, the TiO_2_ nanotube array, and K_2_Ti_6_O_13_-TiO_2_ hybrid nanorod array. ^*^ *p* < .05, ^**^ *p* < .01, ^***^ *p* < .001, ^****^ *p* < .0001 compared with TCP. Data represent the mean ± standard deviation. At least 20 cells were counted. Scale bars in **(A–C)** and **(E–G)** are 40 μm.

In addition to western blotting, immunofluorescence staining was also conducted to determine the protein expression and morphology of differentiated NSCs. Figures 6A–C showed the immunofluorescence staining of Tuj1 and GFAP in NSCs cultured for 7 days. Few of cells cultured on TCPs were Tuj1-positive, and the morphology was clearly spherical. However, more cells expressed Tuj1 when cultured on the TiO_2_ nanotube and K_2_Ti_6_O_13_-TiO_2_ hybrid nanorod arrays. Moreover, NSCs cultured on K_2_Ti_6_O_13_-TiO_2_ hybrid nanorod arrays exhibited stronger fluorescence intensity and more stretching axonal morphology than those cultured on TiO_2_ nanotube arrays. As shown in Figure 6D, the axon lengths among these three groups were ranked as follows: K_2_Ti_6_O_13_-TiO_2_ hybrid nanorod arrays > TiO_2_ nanotube array > TCP. All groups were GFAP-positive and there was no statistically significant difference. The same tendency was observed in the immunofluorescence staining of MAP2 and GFAP after 7 days (Figures 6E–G). Both the fluorescence intensity of MAP2 and proportion of MAP2-positive cells increased in the K_2_Ti_6_O_13_-TiO_2_ hybrid nanorod array group. Furthermore, the extension of the axons of neurons was also significantly enhanced, as shown in Figure 6H, and the average length of the axons was arranged in the following order: K_2_Ti_6_O_13_-TiO_2_ hybrid nanorod arrays (23.77 μm) > TiO_2_ nanotube arrays (15.403 μm) > TCPs (9.055 μm). This indicated that the K_2_Ti_6_O_13_-TiO_2_ hybrid nanorod arrays promoted neural maturation.

Taken together, the K_2_Ti_6_O_13_-TiO_2_ hybrid nanorod arrays could accelerate the differentiation of NSCs without any neural growth or differentiation-inducing factor, which will have great applications in the formation of a fully functional interface between the implant and surrounding tissues.

### Functional Assessment of the Differentiated Neural Stem Cells Induced by Potassium Titanate Nanorod-Decorated Titanium Oxide Hybrid Nanorod Arrays

To achieve better healing of fracture regions, nerve formation in bone fracture areas must be promoted. The incorporation of essential biological elements into the scaffold would be beneficial for fulfilling this target. Incorporating potassium into biomaterials is a feasible strategy to achieve the formation of nerves in newly formed bone areas during metal-bone interface formation.

**FIGURE 7:**
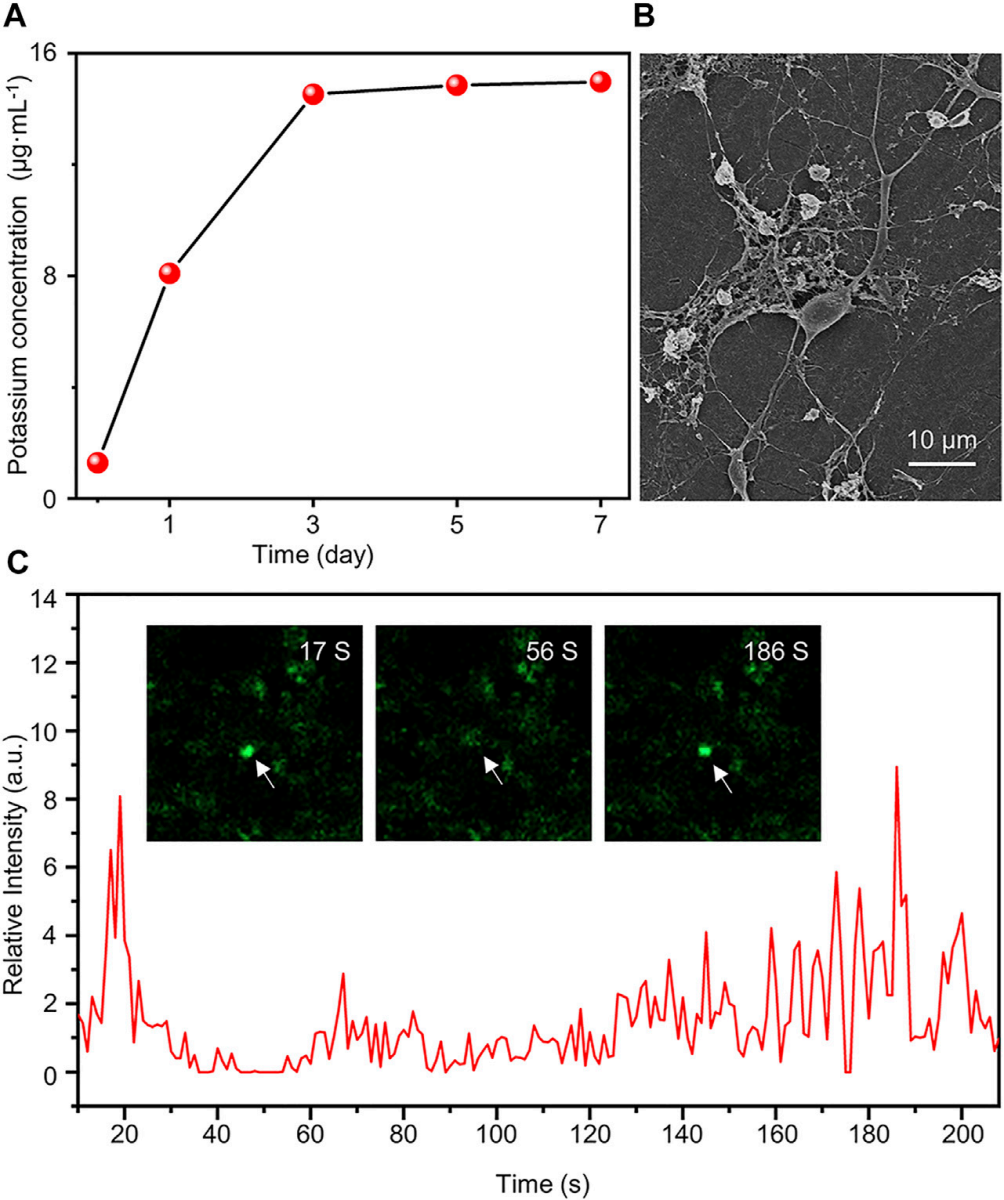
Functional assessment of differentiated cells induced by K_2_Ti_6_O_13_-TiO_2_ hybrid nanorod arrays. **(A)** Concentration of K^+^ in deionized water under different K_2_Ti_6_O_13_-TiO_2_ hybrid nanorod array immersion times. **(B)** SEM image of NSCs after culturing on K_2_Ti_6_O_13_-TiO_2_ hybrid nanorod arrays for 7 days **(C)** Intracellular calcium imaging analysis of NSCs incubated on K_2_Ti_6_O_13_-TiO_2_ hybrid nanorod arrays Insert images are differentiated NSCs cultured on K_2_Ti_6_O_13_-TiO_2_ hybrid nanorod arrays combined with the calcium ion probe fluorescence intensity. The arrow indicates the cell in which the fluorescence intensity changed.

In this study, the K_2_Ti_6_O_13_-TiO_2_ hybrid nanorod arrays may have efficient K^+^ release, contributing to neural differentiation. To verify the K^+^ release of the K_2_Ti_6_O_13_-TiO_2_ hybrid nanorod arrays, inductively coupled plasma-mass spectrometry (ICP-MS) was conducted to quantify the concentration of K^+^ in several samples immersed in H_2_O for 1, 3, 5, and 7 days. Figure 7A shows that the initial concentration of K^+^ was 3.12 μg ml^−1^, which increased with time, reached a peak on the third day, and then remained at this level. After 7 days, 15.1 μg ml^−1^ of K^+^ could be detected in the immersion solution, suggesting that K^+^ could be sustainably released by the K_2_Ti_6_O_13_-TiO_2_ hybrid nanorod arrays and maintained at a certain level. This controllable release was attributed to the nanostructure of the K_2_Ti_6_O_13_-TiO_2_ hybrid nanorod arrays. The potassium titanate nanobelt constructed on the top could rapidly release potassium ions when the K_2_Ti_6_O_13_-TiO_2_ hybrid nanorod arrays were immersed into the cell culture medium. These rapidly released potassium ions were sufficient to accelerate the differentiation of NSCs. Additionally, the K_2_Ti_6_O_13_ nanorods assembled inside the nanotubes sustainably released the ions. Owing to the confinement effect of nanotubes, the K_2_Ti_6_O_13_-TiO_2_ hybrid nanorod arrays could promote the differentiation of neural stem cells continuously without additional supplementation of K^+^.

It has been demonstrated that neuronal cells were induced to express LTP, a persistent increase in synaptic strength, after potassium ion stimulation. K^+^ enhanced the cell membrane potential and activated voltage-gated calcium ion channels, leading to increased intracellular calcium influx. An elevated intracellular Ca^2+^ concentration was conducive to the activation of mitogen-activated protein kinase (MAPK) and calcium/calmodulin-dependent protein kinase (CAMK) pathways, which may contribute to the differentiation of NSCs (Kim et al., 2000).

The activation of the MAPK pathway may he filopodial extension (Pandey and Sharma, 2011). As shown in Figure 7B, SEM images were captured after the cell cultivated for 7 days. The cells exhibited more extended axons and protrusions of dendritic filopodia than those cultured on TCPs (Supplementary Figure S4), which was consistent with a previous report.

Patch-clamp and calcium spark imaging techniques have been widely used to evaluate the function of differentiated cells. Considering the properties of optical opacity, it is difficult to apply the patch-clamp technique in K_2_Ti_6_O_13_-TiO_2_ hybrid nanorod arrays. Thus, the calcium spark imaging technique was employed to evaluate the function of the differentiated NSCs. The NSCs were incubated for 7 days in K_2_Ti_6_O_13_-TiO_2_ hybrid nanorod arrays. The neurotransmitter γ-aminobutyric acid (GABA) was selected to stimulate the NSCs. To track the intracellular change of Ca^2+^, which was labeled with fluorescence, videos were captured, and the time-evolution plot of calcium fluorescence intensity was shown in Figure 7C. After stimulation with GABA, the fluorescence intensity of NSCs was greatly increased and Supplementary movie S1. The fluorescence intensity reached its peak value within 20 s and returned to the resting state value. The peak fluorescence intensity was reached again at 186 s, which was seven times higher than that of the resting state. However, no calcium sparks were detected in the control samples (Supplementary movie S2). These results indicate that differentiated NSCs could respond to GABA stimulation. The abovementioned results demonstrated that K^+^ release from K_2_Ti_6_O_13_-TiO_2_ hybrid nanorod arrays can not only enhance neural differentiation, but also promote the development of neurons and accelerate the maturation of differentiated neurons.

## Conclusion

K_2_Ti_6_O_13_-TiO_2_ hybrid nanorod arrays have been synthesized by hydrothermally assembling K_2_Ti_6_O_13_ nanorods on the channel surface of a TiO_2_ nanotube array. K_2_Ti_6_O_13_-TiO_2_ hybrid nanorod arrays possess multi-functionalized performance for both enhancing the osteogenic differentiation of MSCs and promoting the neural differentiation of NSCs without any growth or differentiation-inducing factors. The osteogenic differentiation ability of the K_2_Ti_6_O_13_-TiO_2_ hybrid nanotube arrays is dominated by the nanotopography of the nanotube array, and the neural differentiation enhancement performance of this hybrid nanotube array is derived from the controlled release of K^+^ in the K_2_Ti_6_O_13_ nanorods inside the TiO_2_ nanotubes. More importantly, the release of K^+^ can also accelerate the maturity of differentiated neurons and appear to have ideal electrophysiological properties within only 7 days. The promoted osteogenic/neural differentiation derived from K_2_Ti_6_O_13_-TiO_2_ hybrid nanorod arrays suggested the designed materials show promise in forming neural network reconstruction in the bone-implant interface, which can improve the ability of repairing bone defect.

## Data Availability

The original contributions presented in the study are included in the article/Supplementary Material, further inquiries can be directed to the corresponding authors.
