# Supplementary material for: Potassium Titanate Assembled Titanium Dioxide Nanotube Arrays Endow Titanium Implants Excellent Osseointegration Performance and Nerve Formation Potential
Source: Front Chem. 2022 Jan 25;10:839093. doi: 10.3389/fchem.2022.839093 (PMC8821153; doi:10.3389/fchem.2022.839093)
Supplement: Supplementary file 3 [file DataSheet1.docx]

Supplementary Materials

**
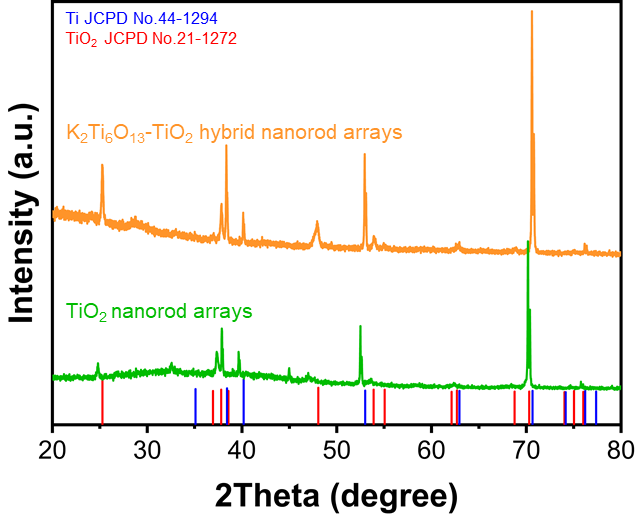
**

**Supplementary Fig. 1** XRD pattern of K_2_Ti_6_O_13_-TiO_2_ hybrid nanorod arrays and TiO_2_ nanotube arrays.

XRD was employed to characterize the crystalline structures of the resultant samples. All the diffraction peaks of the sample can be easily indexed to anatase TiO_2_ and Ti, corresponding to JCPD cards #21-1272 and #44-1294, respectively. After hydrothermal treatment, although almost all of the peaks in the XRD image still belonged to TiO_2_ and titanium, a very broad peak could be found at approximately 28°, which is the peak of K_2_Ti_6_O_13._


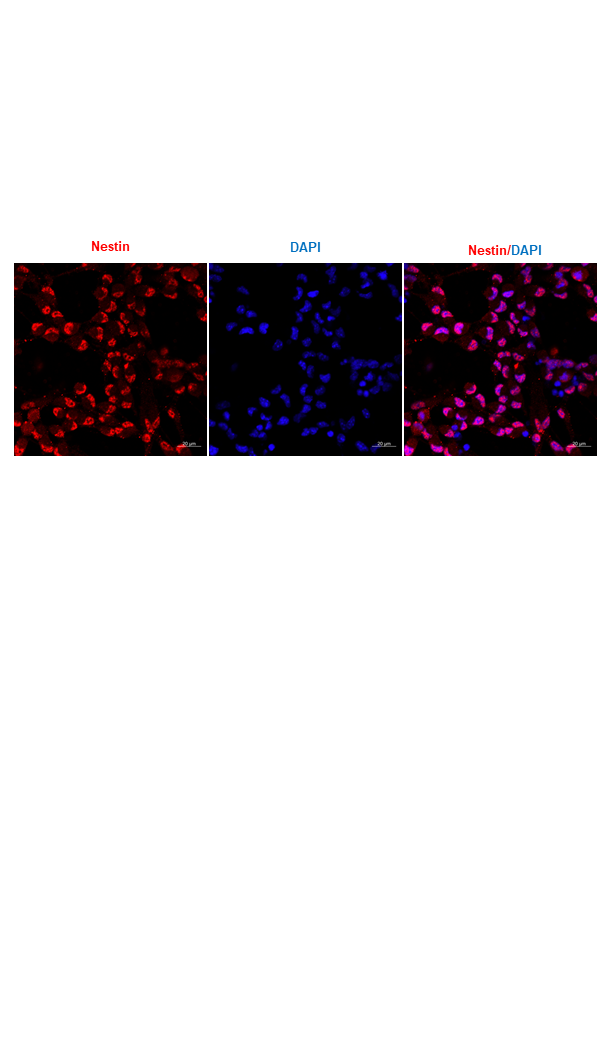


**Supplementary Fig. 2** Immunofluorescence cell staining of stemness marker Nestin and DAPI. The mNSCs were cultured on culture plate.

Nestin immunofluorescence staining was used to assess the quality of NSCs. Almost all of the NSCs cultured on the culture plate were nestin-positive, indicating the high multi-differential potential of the isolated NSCs.


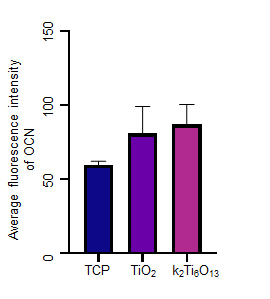


**Supplementary Fig. 3** Statistical analysis of the average OCN fluorescence intensity using ImageJ software.


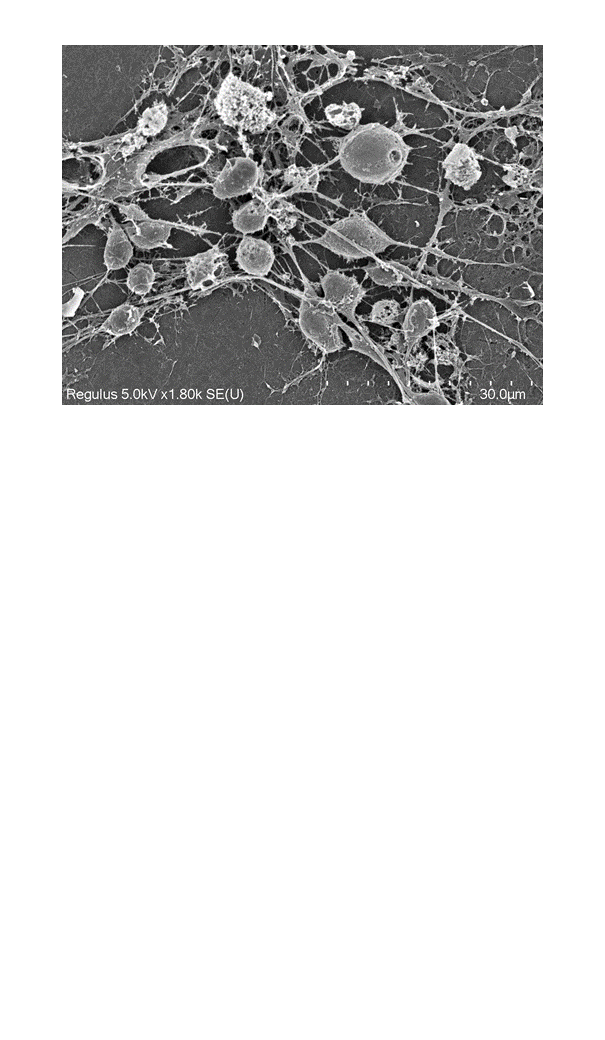


**Supplementary Fig. 4** SEM image of mNSCs cultured on tissue culture plate for 7 d. The scale bar represents 15 μm. The cells cultured on tissue culture plates exhibited less extended axons and protrusions of dendritic filopodia.

**Supplementary Table 1:** Sequences of RT-PCR primers.

| Gene | Forward primers (5’-3’) | Reverse primers (5’-3’) |
| --- | --- | --- |
| β-actin | GGCTGTATTCCCCTCCATCG | CCAGTTGGTAACAATGCCATGT |
| Nestin | TGCCCTAGAGACGGTGTCTCA | AATCGCTTGACCTTCCTCCC |
| Tuj1 | TATGAAGATGATGACGAGGAATCG | TACAGAGGTGGCTAAAATGGGG |
| GFAP | CCAAGCCAAACACGAAGCTAA | CATTTGCCGCTCTAGGGACTC |
| MAP2 | GCCAGCCTCAGAACAAACAG | AAGGTCTTGGGAGGGAAGAAC |

| Gene | Forward primers (5’-3’) | Reverse primers (5’-3’) |
| --- | --- | --- |
| GAPDH | GCCTCGTCTCATAGACAAGATGGT | GAAGGCAGCCCTGGTAACC |
| Runx2 | AATGCCTCCGCTGTTATG | TTCTGTCTGTGCCTTCTTG |
| OPN | TCCTGTCTCCCGGTGAAAGT | GGCTACAGCATCTGAGTGTTTGC |
| OCN | AAGCCCAGCGACTCTGAGTCT | CCGGAGTCTATTCACCACCTTACT |

**Supplementary Table 2:** Sequences of RT-PCR primers.

Supplementary Movie 1. The NSCs, seeded on K_2_Ti_6_O_13_-TiO_2_ hybrid nanorod arrays for 7 days, are labeled with Fluo-4 AM, stimulated by GABA, and visualized using a confocal microscope.

Supplementary Movie 2. The NSCs, seeded on TCPs for 7 days, are labeled with Fluo-4 AM, stimulated by GABA, and visualized using a confocal microscope.
